# Fluorofenidone Alleviates Renal Fibrosis by Inhibiting Necroptosis Through RIPK3/MLKL Pathway

**DOI:** 10.3389/fphar.2020.534775

**Published:** 2020-12-16

**Authors:** Qin Dai, Yan Zhang, Xiaohua Liao, Yupeng Jiang, Xin Lv, Xiangning Yuan, Jie Meng, Yanyun Xie, Zhangzhe Peng, Qiongjing Yuan, LiJian Tao, Ling Huang

**Affiliations:** ^1^ Department of Nephrology, Xiangya Hospital, Central South University, Changsha, China; ^2^ Department of Respirology, Xiangya Hospital, Central South University, Changsha, China

**Keywords:** fluorofenidone, necroptosis, receptor-interacting protein kinase 3-mixed lineage kinase domain-like protein pathway, inflammation, renal fibrosis

## Abstract

Cell death and sterile inflammation are major mechanisms of renal fibrosis, which eventually develop into end-stage renal disease. “Necroptosis” is a type of caspase-independent regulated cell death, and sterile inflammatory response caused by tissue injury is strongly related to necrosis. Fluorofenidone (AKF-PD) is a novel compound shown to ameliorate renal fibrosis and associated inflammation. We investigated whether AKF-PD could alleviate renal fibrosis by inhibiting necroptosis. Unilateral ureteral obstruction (UUO) was used to induce renal tubulointerstitial fibrosis in C57BL/6J mice. AKF-PD (500 mg/kg) or necrostatin-1 (Nec-1; 1.65 mg/kg) was administered simultaneously for 3 and 7 days. Obstructed kidneys and serum were harvested after euthanasia. AKF-PD and Nec-1 ameliorated renal tubular damage, inflammatory-cell infiltration, and collagen deposition, and the expression of proinflammatory factors (interlukin-1β, tumor necrosis factor [TNF]-α) and chemokines (monocyte chemoattractant protein-1) decreased. AKF-PD or Nec-1 treatment protected renal tubular epithelial cells from necrosis and reduced the release of lactate dehydrogenase in serum. Simultaneously, production of receptor-interacting protein kinase (RIPK)3 and mixed lineage kinase domain-like protein (MLKL) was also reduced 3 and 7 days after UUO. AKF-PD and Nec-1 significantly decreased the percentage of cell necrosis, inhibiting the phosphorylation of MLKL and RIPK3 in TNF-α- and Z-VAD–stimulated human proximal tubular epithelial (HK-2) cells. In conclusion, AKF-PD and Nec-1 have effective anti-inflammatory and antifibrotic activity in UUO-induced renal tubulointerstitial fibrosis, potentially mediated by the RIPK3/MLKL pathway.

## Introduction

Chronic kidney disease (CKD) is a public health concern due to its high morbidity and increasing economic burden. CKD affects 10–15% of the global population and is responsible for one million deaths worldwide (Levin et al., 2017).

Renal fibrosis is the final common pathway of CKD of virtually any cause and manifests as tubulointerstitial fibrosis and glomerulosclerosis. Renal tubulointerstitial fibrosis is associated with inflammation, cell death, proliferation of the extracellular matrix, and endothelium–mesenchymal transition. However, the underlying molecular mechanisms have not been identified and a valid therapeutic approach is lacking (Zhang et al., 2009; Boor et al., 2010; Grenier, 2011).

Tissue damage and sterile inflammation are important triggers for the progression of renal interstitial fibrosis. Following injury, necrotized cells release danger-associated molecular patterns (DAMPs), which usually contribute to the sterile inflammatory responses related to necrosis (Martin Seamus, 2016).

“Necroptosis” is a novel, caspase-independent type of regulated cell death. Necroptosis involves receptor-interacting protein kinase (RIPK)1, RIPK3, and mixed lineage kinase domain-like protein (MLKL) and is mediated by the formation of the RIPK1/RIPK3 necrosome (Cho et al., 2009; Sun et al., 2012). Upon phosphorylation of RIPK3 by RIPK1, phosphorylated RIPK3 activates MLKL, which eventually results in plasma membrane rupture and releases proinflammatory cellular contents to the extracellular space (Wallach and Kang, 2018). It has been reported that RIPK3/MLKL-mediated necroptosis contributes to interstitial fibrosis related to tubulointerstitial injury (Xiao et al., 2017; Popper et al., 2019) and ischemia–reperfusion injury in the kidney (Linkermann et al., 2013). Moreover, the RIPK1 inhibitor necrostatin-1 (Nec-1) has an established role in inhibiting necroptosis (Degterev et al., 2005; Degterev et al., 2008) in ischemia–reperfusion injury in the kidney (Linkermann et al., 2013).

Fluorofenidone (1-[3-fluorophenyl]-5-methyl-2-[1H]-pyridone), which is usually abbreviated as AKF-PD, is a newly developed drug. It can attenuate fibrosis in the liver, lung, heart, and kidney (Chen et al., 2012; Peng et al., 2014; Tang et al., 2015; Song et al., 2016; Chen et al., 2019). AKF-PD is in phase-I clinical trials in China. Studies have shown that AKF-PD can inhibit inflammation in renal fibrosis (Tang et al., 2015). However, the role of AKF-PD in the relationship between inflammation and necroptosis in renal tubulointerstitial fibrosis is not known.

In this study, we aimed, for the first time, to illustrate the role of AKF-PD in renal inflammation in renal tubulointerstitial fibrosis after unilateral ureteral obstruction (UUO) in mice.

## Materials and Methods

### Ethical Approval of the Study Protocol

This study protocol was approved by the Ethics Review Committee for Animal Experimentation of Central South University.

### Reagents and Antibodies

Human proximal tubular epithelial (HK-2) cells were purchased from the Medical Science Experimentation Center of Sun Yat-sen University (Guangzhou, China). Nec-1 (catalog number S8073) and Z-VAD-FMK (S7023) were obtained from Selleck Chemicals (Houston, TX, United States). Recombinant human tumor necrosis factor-alpha (TNF-α) was purchased from R&D Systems (210-TA; Minneapolis, MN, United States). Mouse interleukin (IL)-1β (88-7013-88) and TNF-α (88-7324-88) ELISA kit were provided by ThermoFisher Scientific (MA, United States). Quantitative polymerase chain reaction (qPCR) primers for IL-1β, TNF-α, and monocyte chemoattractant protein (MCP)-1 were purchased from Generay Biotech (Shanghai, China). A lactate dehydrogenase (LDH) assay kit was obtained from Jiancheng (A020-2-2; Nanjing, China). The FITC Annexin V Apoptosis Detection Kit I (556547) was purchased from BD Pharmingen (United Kingdom). The primary antibody of RIPK3 was purchased from NOVUS (NBP1-77299SS; Saint Louis, MO, United States). Mouse MLKL was obtained from Millipore (MABC604; Bedford, MA, United States). Human MLKL (ab184718) and phosphorylated S358 MLKL (ab187091) were purchased from Abcam (Cambridge, United Kingdom) and F4/80 from Novus Biologicals (#CI-A3-1; Minneapolis, United States). Primary and secondary antibodies against anti-glyceraldehyde-3-phosphate dehydrogenase (GAPDH) were purchased from Sigma-Aldrich (#G9295; Darmstadt, Germany).

### Animals

Male C57BL/6J mice (6–8 weeks old) were purchased from the Silaike Laboratory Animal Center (Shanghai, China). Mice were maintained in a standard laboratory environment with a 12-h light–dark cycle with unrestricted access to water and chow.

Mice were assigned randomly to four groups of six: sham-operated, UUO, UUO + AKF-PD, and UUO + Nec-1. UUO mice were induced according to a method described previously (Ren et al., 2015). In brief, a 1- to 1.5-cm longitudinal incision was made about 0.25 cm above the left costal ridge and 0.5 cm on the left side of the spine; then, the abdominal cavity was opened layer by layer, to expose the kidney. Part of the renal capsule and adipose tissue at the lower pole and renal pedicle of the kidney were stripped, separating the ureter. UUO was induced by ligation of the left ureter of the mouse. One day after undergoing ligation, mice were administered AKF-PD (500 mg/kg/day, 0.5% carboxymethyl cellulose sodium as the solvent) or Nec-1 (1.65 mg/kg/d, DMSO, PEG300 as the solvent) by intraperitoneal injection. The sham group and the UUO group were administered 0.5% carboxymethyl cellulose sodium (i.g.) simultaneously.

Mice were sacrificed at 3 and 7 days after UUO. Blood samples were collected through cardiac puncture for LDH measurement. Part of the left kidney was fixed in 10% neutral-buffered formalin and 2.5% glutaraldehyde phosphate buffer (pH 7.4), respectively, for pathology, immunohistochemical (IHC) analyses, and morphological examination of renal tissue. The remaining kidney was preserved in liquid nitrogen for qPCR and Western blotting.

### Renal Pathology and IHC Analyses

Formalin-fixed kidneys were embedded in paraffin. Paraffin-embedded tissues were sliced into sections 4 µm thick. Staining (hematoxylin and eosin [H&E], Masson trichrome) was conducted to evaluate tubulointerstitial injury and collagen deposition, which were graded as described previously (Radford et al., 1997; Li et al., 2011). An EnVision™ System (Dako Diagnostics, Zug, Switzerland) was used for IHC staining of histology sections using 3,3′-diaminobenzidine. Antigen retrieval was achieved using a microwave oven, and slides were incubated with a primary antibody against F4/80 (1:100 dilution). Positive staining in the renal cortex was measured using computerized morphometry (ImagePro Plus 6.0 software, Media Cybernetics, Bethesda, MD, United States) in randomly selected fields at ×200 magnification. The analyses mentioned above were conducted in a double-blind manner by two individual pathologists as previously described (Broekema et al., 2007).

### Transmission Electron Microscopy

Renal tissue fragments were fixed in 2.5% glutaraldehyde phosphate buffer (pH 7.4) followed by 1% osmium tetroxide. After dehydration by a graded series of alcohol solutions, renal tissue was embedded in epoxy resin. Then, uranyl acetate and lead citrate were used to stain the sections. Samples were examined by a digital electron microscope. Transmission electron microscopy (TEM) images showed necrosis of tubular epithelial cells, identified by nuclear chromatin irregularities, ruptured plasma membranes, and cytoplasm and organelle expulsion.

### LDH Assay

Plasma was separated from blood samples by centrifugation. The LDH level was measured according to the protocol of the LDH assay kit.

### Real-Time PCR

We isolated total RNA from kidney tissue using TRIzol^®^ Reagent (Life Technologies, Carlsbad, CA, United States) according to the manufacturer instructions. A volume corresponding to 1 μg of the total RNA was used to synthesize complimentary DNA employing the RevertAid™ First Strand cDNA Synthesis kit (Life Technologies). The specific primers were synthesized by Sangon Biotech (Shanghai, China) according to GenBank data. The primer sequences (forward and reverse, respectively) were 5ʹ-CTG​GTG​TGT​GAC​GTT​CCC​AT-3ʹ and 5ʹ-TCG​TTG​CTT​GGT​TCT​CCT​TGT-3ʹ for IL-1β; 5ʹ-CAC​CAC​GCT​CTT​CTG​TCT​ACT-3ʹ and 5ʹ-AAC​TGA​TGA​GAG​GGA​GGC​CAT-3ʹ for TNF-α; 5ʹ-TTA​AAA​ACC​TGG​ATC​GGA​ACC​AA-3ʹ and 5ʹ-GCA​TTA​GCT​TCA​GAT​TTA​CGG​T-3ʹ for MCP-1; and 5ʹ-CAC​TGT​CGA​GTC​GCG​TCC-3ʹ and 5ʹ-TCA​TCC​ATG​GCG​AAC​TGG​TG-3ʹ for β-actin.

mRNA expression was measured using the SYBR^®^ Green qPCR kit (Thermo Scientific) in the CFX96 real-time detection system (Bio-Rad Laboratories, Hercules, CA, United States). mRNA expression in each sample was normalized to that of β-actin mRNA. Statistical analyses were conducted using the comparative 2-ΔΔCT method.

### ELISA

Kidney protein was extracted from tissue homogenate (l% Nonidet P-40, 50 mM HEPES, l mM PMSF, and PBS 1 mol/L). Levels of IL-1β and TNF-α were measured according to the protocol of the ELISA kit.

### Cell Culture

HK-2 cells were cultured in Dulbecco’s modified Eagle’s medium (DMEM)/F12 (Gibco, Billings, MT, United States) containing penicillin (100 U/ml; Gibco), streptomycin (100 μg/ml; Gibco), and 10% fetal bovine serum (FBS, Gibco) in an atmosphere of 5% CO_2_ at 37°C.

HK-2 cells were seeded into 12-well culture plates for 24 h with DMEM/F12 with 10% FBS, which was then replaced with DMEM/F12 with 2% FBS for 12 h. HK-2 cells were exposed to 50 ng/ml TNF-α, 30 uM Z-VAD for 6 h after pretreatment with AKF-PD (400 μg/ml) for 24 h. Cells in the Nec-1 group were exposed to Nec-1 (50 µM) for 6 h. Whole cell proteins were harvested for Western blotting.

### Flow Cytometry

Annexin V-FITC/propidium iodide (PI) was used to stain HK-2 cells to detected cell necrosis. Briefly, cells were washed twice with cold PBS and then resuspended in 1X binding buffer. FITC annexin V and PI were added and the cells incubated for 15 min at room temperature (25°C) in the dark. Staining was then assessed via flow cytometry (BD FACSCanto II, United Kingdom).

### Western Blotting

Kidney tissue and cell proteins were extracted using sodium dodecyl sulfate lysis buffer. The BCA protein assay kit was used to measure protein concentration. Then, proteins were separated by sodium dodecyl sulfate-polyacrylamide gel electrophoresis using 8–12% gels based on the molecular weight of the target protein. Proteins were transferred onto polyvinylidene difluoride (PVDF) membranes (Millipore) under a constant current. PVDF membranes were blocked with 5% skimmed milk powder dissolved in 1 × TBS-T [20 mmol/L Tris-HCl, 150 mmol/L NaCl (pH 7.6), and 0.05% Tween-20] for 1 h at room temperature. Then, PVDF membranes were incubated with primary antibodies against RIPK3 (1:1,000 dilution), MLKL (1:1,000), phosphorylated (*p*)-MLKL (1:1,000), and GAPDH (1:10,000) overnight at 4°C. Next, PVDF membranes were incubated with horseradish peroxidase–conjugated secondary antibodies for 1 h at room temperature after washing with 1× TBST. Signals were visualized by enhanced chemiluminescence reagents (GE Healthcare, Little Chalfont, United Kingdom) and bands were quantified using ImageJ (National Institutes of Health, Bethesda, MD, United States).

### Statistical Analyses

Data are represented as mean ± standard deviation (SD). SPSS v25.0 (IBM, Armonk, NY, United States) was used for statistical analyses. Comparisons among groups were analyzed with one-way ANOVA; each experiment was repeated at least in triplicate. A *p*-value < 0.05 was considered significant.

## Results

### AKF-PD Ameliorated Renal Tubulointerstitial Injury and Fibrosis in UUO Mice

**FIGURE 1:**
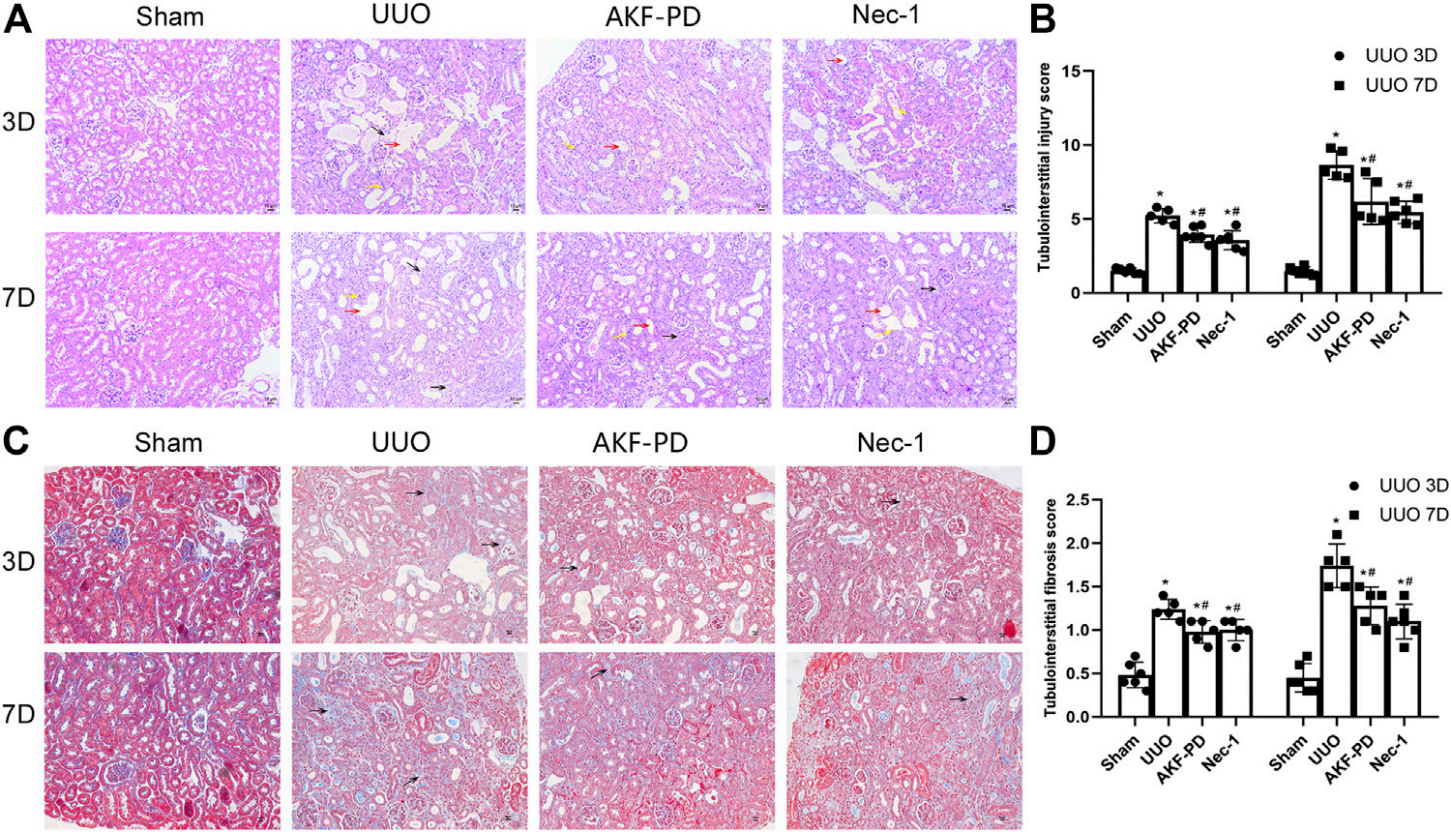
AKF-PD and Nec-1 attenuated the histological changes in the obstructed kidney after UUO on days 3 and 7 . **(A)**, **(B)** AKF-PD and Nec-1 attenuated tubulointerstitial injury in UUO on days 3 and 7 (×200) (H&E staining of kidney sections, ×200). The red arrow points to the dilated area of the renal tubule; the yellow arrow points to the infiltration area of inflammatory cells; the black arrow points to the renal interstitial fibrosis area. **(C)**, **(D)** AKF-PD and Nec-1 alleviated extracellular matrix deposition in UUO on days 3 and 7 (Masson’s trichrome staining of kidney sections, ×200). All data are presented as means ± SD, *n* ≥ 5; *p < 0.05 versus sham, #*p* < 0.05 versus UUO. Data were analyzed by one-way ANOVA. AKF-PD, fluorofenidone; Nec-1, necrostatin-1; UUO, unilateral ureteral obstruction.

To determine the efficacy of AKF-PD and Nec-1 against renal fibrosis, mice were subjected to UUO, a classic experimental model used to induce renal interstitial fibrosis (da Silva Andrei et al., 2015). H&E staining showed renal tubulointerstitial damage in mice at 3 and 7 days after UUO (Figure 1A). This manifested as tubule expansion, destruction of renal tubular cells, and infiltration of inflammatory cells. Masson trichrome staining revealed collagen deposition (Figure 1C). Compared with the sham-treated group, treatment with AKF-PD or Nec-1 significantly attenuated renal tubulointerstitial damage and collagen deposition at 3 and 7 days after UUO (Figure 1B,D). These results demonstrated that AKF-PD exerted an antifibrotic effect in the UUO model, which is consistent with our previous studies (Tang et al., 2015). Nec-1 (a small-molecule inhibitor of necroptosis) also significantly reduced renal damage and collagen deposition after UUO. These data suggest that necroptosis inhibition may have a positive effect against renal fibrosis induced by UUO.

### AKF-PD Reduced the UUO-Induced Expression of Proinflammatory Factors and Chemokines and Macrophage Infiltration

**FIGURE 2:**
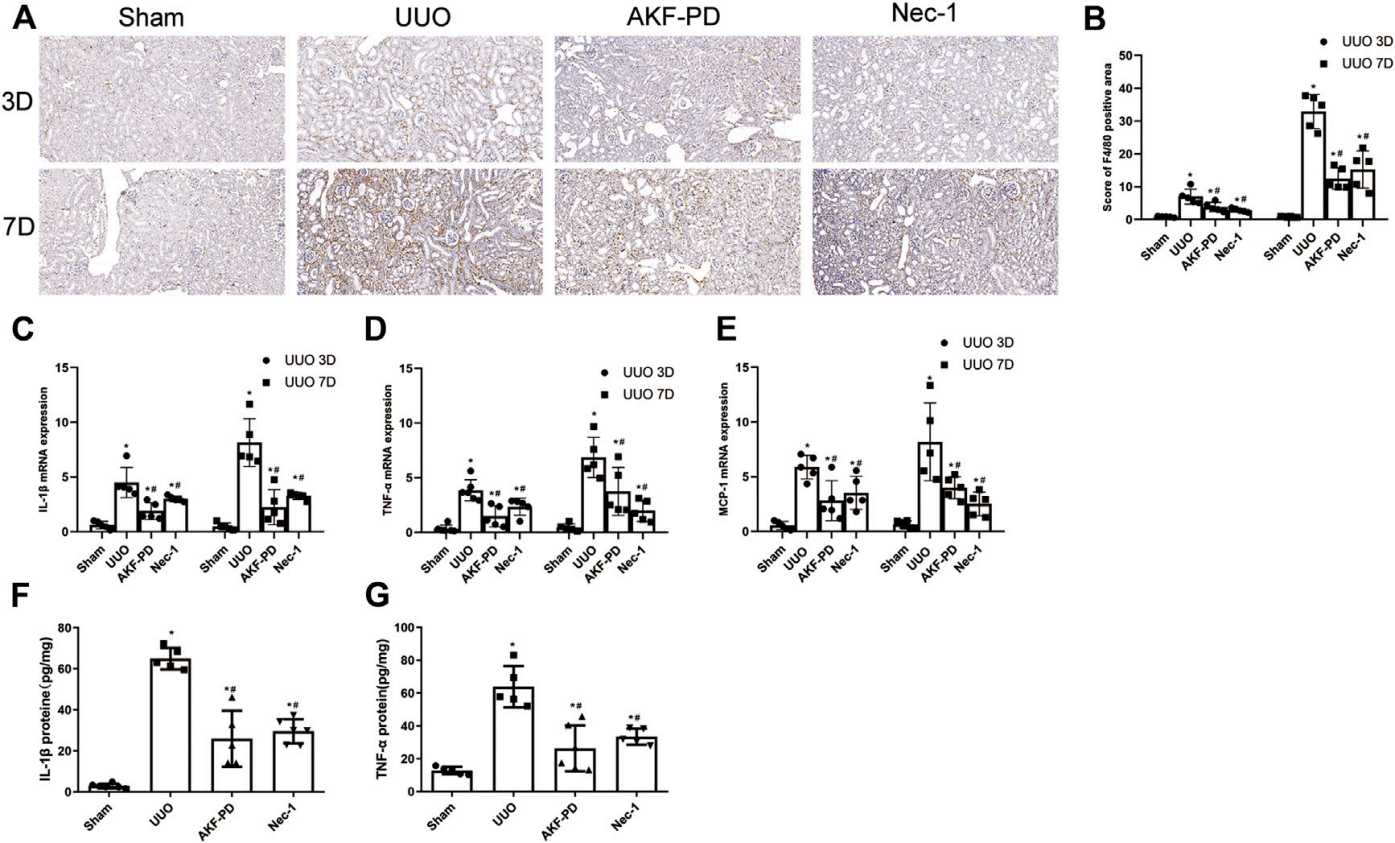
AKF-PD and Nec-1 reduced the infiltration of macrophage and the expression of inflammatory factors and chemokines in UUO. **(A)**, **(B)** AKF-PD and Nec-1 affected F4/80 protein expression in the obstructed kidneys stained by immunohistochemistry (×200). **(C)** IL-1β, **(D)** TNF-α and MCP-1, **(E)** mRNA expression in the kidneys, **(F)** IL-1β protein expression in UUO kidneys on days 3. **(G)** TNF-α protein expression in UUO kidneys on day 3. All data are presented as means ± SD, *n* ≥ 5. *p < 0.05 versus sham, #*p* < 0.05 versus UUO. Data were analyzed by one-way ANOVA. AKF-PD, fluorofenidone; Nec-1, necrostatin-1; UUO, unilateral ureteral obstruction.

Macrophage infiltration is a major driver of progression to renal interstitial fibrosis (Chevalier Robert et al., 2009). Expression of proinflammatory factors and chemokines is crucial for macrophage recruitment. IHC staining of F4/80 was undertaken to examine macrophage infiltration. Macrophage infiltration increased in the kidneys of UUO mice in a time-dependent manner and was reduced by treatment with AKF-PD or Nec-1 (Figure 2A,B). Expression of proinflammatory factors (IL-1β and TNF-α) and a chemokine (MCP-1) in the kidney was increased dramatically in UUO mice at the transcriptional and protein levels according to RT-PCR and ELISA results. Administration of AKF-PD or Nec-1 significantly inhibited the production of proinflammatory factors and chemokines at the transcriptional and protein level (Figure 2C–G).

### AKF-PD Alleviated Cell Necrosis in UUO

**FIGURE 3:**
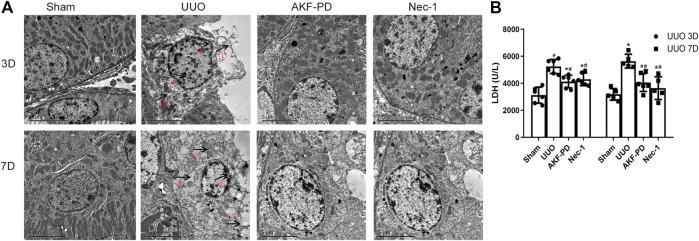
AKF-PD and Nec-1 alleviate cell necrosis in UUO. **(A)** Cell morphology of renal tissue observed by transmission electron microscopy (×5,000). ①: Cell membrane perforation or deformation; ②: nuclear membrane prolapse or rupture; ③: cytoplasmic transparency; ④: chromatin condenses into irregular plaque; ⑤: organelle swelling. **(B)** The level of the released LDH in the serum. All data are presented as means ± SD, *n* ≥ 5. *p < 0.05 versus sham, #*p* < 0.05 versus UUO. Data were analyzed by one-way ANOVA. AKF-PD, fluorofenidone; Nec-1, necrostatin-1; UUO, unilateral ureteral obstruction; LDH, lactate dehydrogenase.

Upon tissue damage, tubular cell necrosis is triggered by inflammation in UUO (Chevalier Robert et al., 2009; Pooper et al., 2019). Necrosis is manifested mainly as nuclear lysis, swelling, disintegration of organelles, rupture of the cell membrane, release of many proinflammatory cell contents (including DMAPS), and then activation of the innate immune response (Kono et al., 2014). To determine whether AKF-PD or Nec-1 was involved in the death of tubular cells after UUO, TEM was used to analyze the death of renal tubular epithelial cells in the kidney. TEM revealed that in UUO mice, tubular epithelial cells often exhibited ultrastructural signs of necroptosis, such as increased volume, disordered intracellular structure, nuclear chromatin irregularities, organelle swelling, and ruptured plasma membranes, different from other regulated cell death (including apoptosis and ferroptosis). UUO mice treated with AKF-PD or Nec-1 improved the ultrastructural features of cell necrosis (Figure 3A). Furthermore, we measured the LDH in serum upon necrosis. The AKF-PD group and the Nec-1 group had decreased release of LDH at 3 and 7 days after UUO. These findings suggested that AKF-PD could protect renal tubular epithelial cells from necrosis and could be considered an inhibitor of necroptosis.

### AKF-PD Reduced Expression of RIPK3 and MLKL in the UUO Kidney Model

**FIGURE 4:**
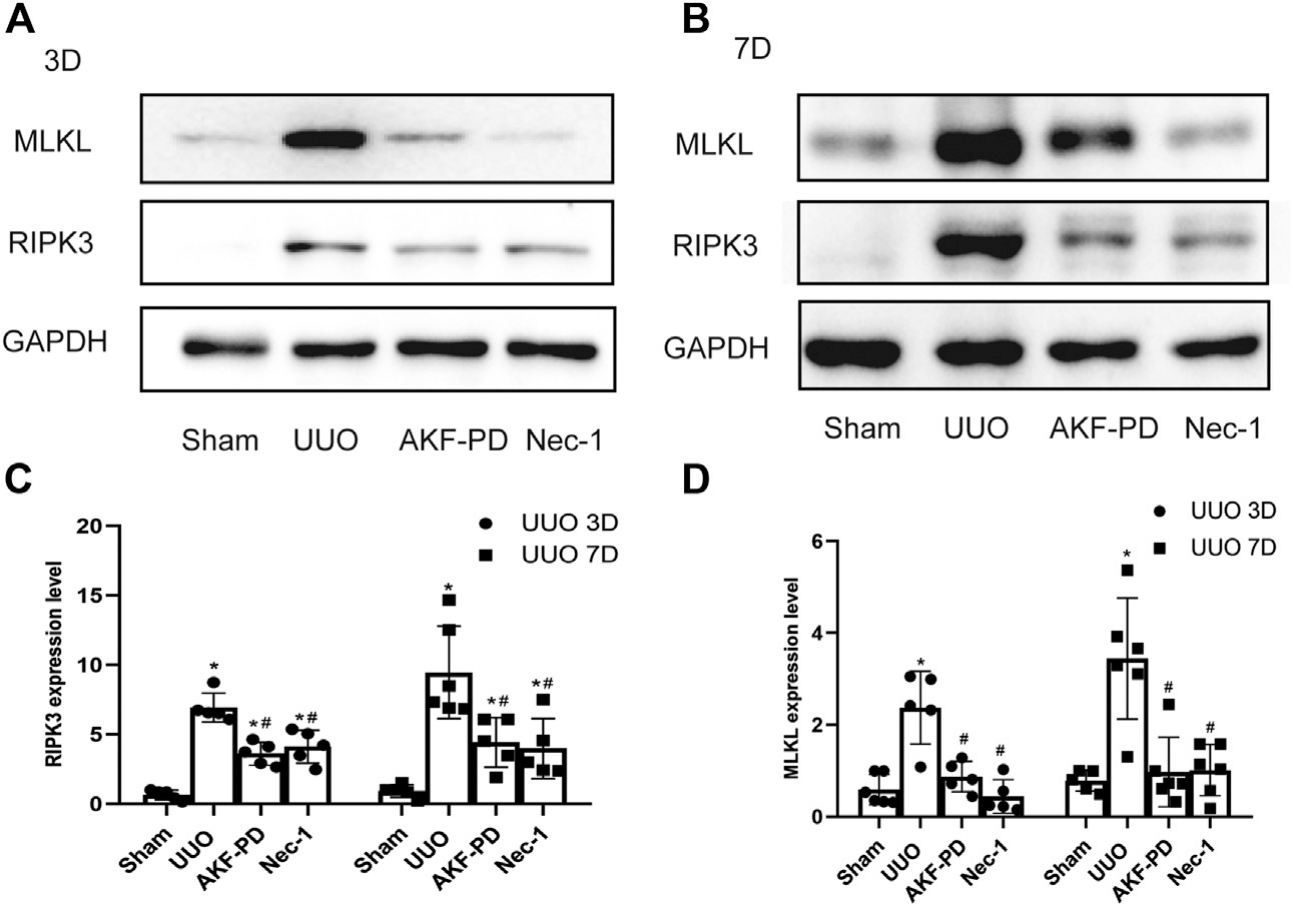
AKF-PD and Nec-1 inhibited the expression of RIPK3 and MLKL in UUO. **(A)**–**(D)** The expression of RIPK3 and MLKL in UUO kidneys on days 3 and 7 detected by Western blots. All data are presented as means ± SD, *n* ≥ 5. *p < 0.05 versus sham, #*p* < 0.05 versus UUO. Data were analyzed by one-way ANOVA. AKF-PD, fluorofenidone; Nec-1, necrostatin-1; UUO, unilateral ureteral obstruction; RIPK, receptor-interacting protein kinase; MLKL, mixed lineage kinase domain-like protein.

To further explore the mechanism of action of AKF-PD on necrosis, we focused on necroptosis. The protein levels of RIPK3 and MLKL in kidney tissue were measured by Western blotting. Protein expression of RIPK3 and MLKL in the kidney was upregulated significantly at 3 and 7 days after UUO. Kidneys treated with AKF-PD or Nec-1 showed reduced expression of RIPK3 and MLKL compared to kidneys of UUO-induced mice (Figure 4A–D). These results suggested that AKF-PD protected renal tubular epithelial cells from necroptosis *via* the RIPK3/MLKL pathway.

### AKF-PD Inhibited Necroptosis Through the RIPK3/MLKL Pathway *In Vitro*

**FIGURE 5:**
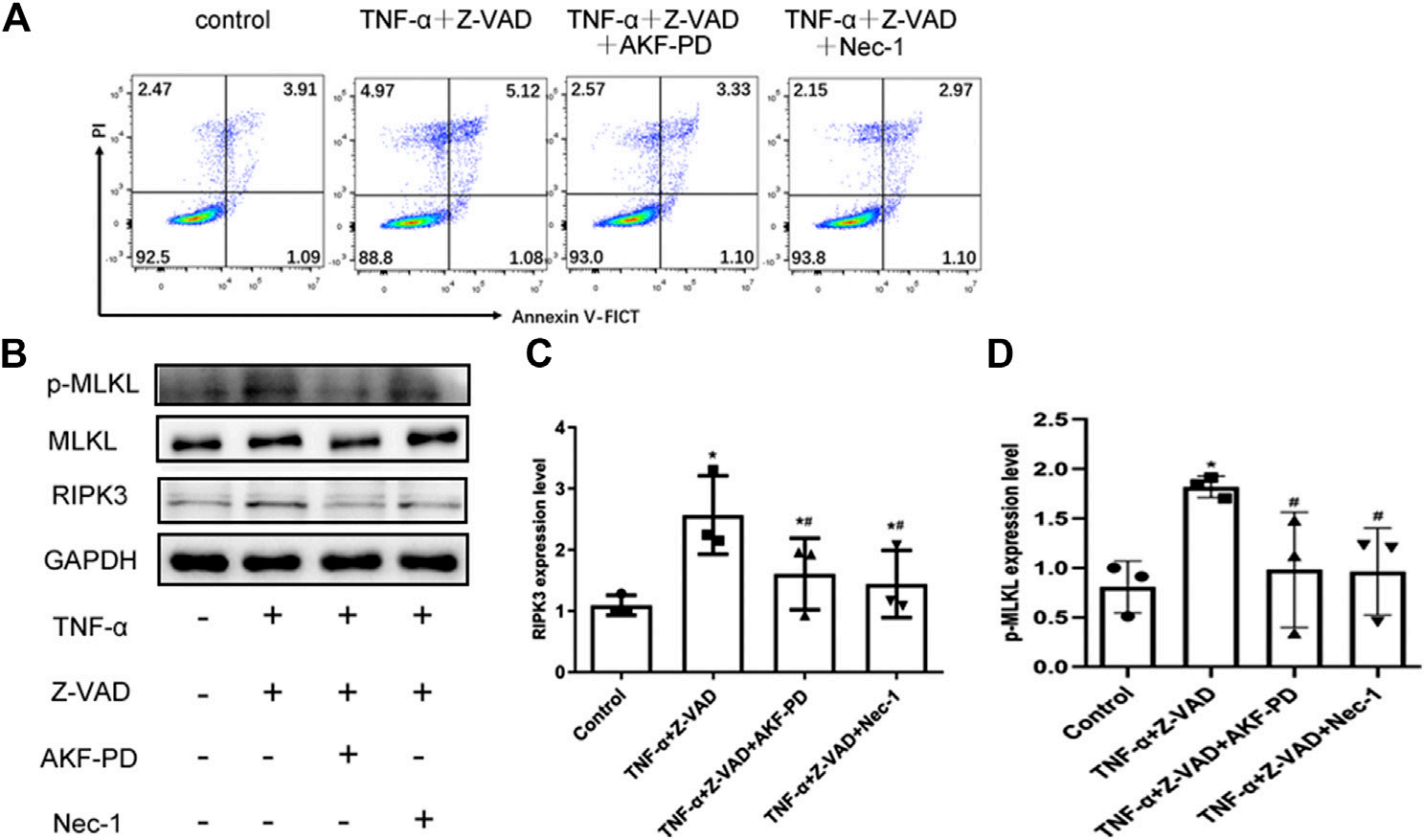
AKF-PD and Nec-1 inhibited RIPK3- and *p*-MLKL–related necroptosis. **(A)** Percentages of necrotic cells determined by FACS analysis using annexin V-FITC and PI. **(B)**–**(D)** The expression of RIPK3, MLKL, and *p*-MLKL stimulated with TNF-α and Z-VAD in HK-2 cells detected by Western blots. All data are presented as means ± SD, *n* = 3. *p < 0.05 versus control, #*p* < 0.05 versus TNF-α+Z-VAD group. Data were analyzed by one-way ANOVA. AKF-PD, fluorofenidone; Nec-1, necrostatin-1; RIPK, receptor-interacting protein kinase; MLKL, mixed lineage kinase domain-like protein.

To further clarify the effects of AKF-PD on necroptosis, we stimulated necroptosis of human renal tubular epithelial (HK-2) cells by treatment with TNF-α and Z-VAD. Treatment with AKF-PD or Nec-1 decreases the percentage of annexin V (-)/PI(+) HK-2 cells (Figure 5A), and protein expression of RIPK3 and P-MLKL also decreased (Figure 5B–D). This result further supported the notion that AKF-PD could suppress RIPK3-mediated necroptosis effectively.

## Discussion

Our findings demonstrated that AKF-PD treatment could significantly mitigate renal tubular injury, macrophage infiltration, and collagen deposition in the UUO-induced mouse model, observations that were consistent with our previous studies (Tang et al., 2015). We provide evidence that necroptosis plays a role in UUO progression *in vivo* as well as in TNF-α/Z-VAD–stimulated necroptosis in HK-2 cells *in vitro*. Herein we provide, for the first time, evidence that (1) blockade of necroptosis with Nec-1 or AKF-PD can attenuate renal injury, inflammatory responses, and renal fibrosis in UUO mice and (2) the underlying mechanism seems to involve inhibition of the RIPK1/RIPK3/MLKL pathway.

AKF-PD has a chemical structure similar to that of pirfenidone. AKF-PD is a novel low-molecular-weight compound developed by Central South University (Changsha, Hunan Province, China). Previously, we showed that AKF-PD inhibited TGF-β expression and reduced the production of ROS more potently than pirfenidone (PD); the safety of AKF-PD was also better than that of PD (Declèves and Sharma, 2010; Lv et al., 2015). Our previous studies suggested that AKP-PD had therapeutic effects in various experimental models of fibrosis in the liver, kidney, and lung (Peng et al., 2014; Tang et al., 2015; Song et al., 2016). Furthermore, we previously suggested that AKF-PD could inhibit oxidative stress, inflammation, fibroblast activation, apoptosis, and transdifferentiation of renal tubular epithelial cells, but the antifibrotic mechanism and target of AKF-PD were not identified.

UUO has been used to elucidate the pathogenesis of obstructive nephropathy and progressive nephropathy (Klahr and Jeremiah, 2002; Vaughan et al., 2004). The pathogenesis of renal fibrosis following UUO includes interstitial infiltration of macrophages, tubular cell death, and phenotypic transition of resident renal cells (Zhang et al., 2009; Boor et al., 2010; Grenier, 2011). Ureteral ligation triggers cell death and inflammation. Apoptosis and antiapoptotic signaling in the antifibrotic mechanism of AKF-PD have been studied by our group (Ning et al., 2011). However, whether necroptosis is present in CKD progression is not known, and researchers have not investigated whether AKF-PD can improve necroptosis in CKD progression.

Our study provides evidence suggesting that necroptosis occurs in renal tubular epithelial cells at the early stage of UUO, data that are consistent with results from other researchers (Xiao et al., 2017). Moreover, we showed, for the first time, that treatment with AKF-PD or Nec-1 reduced necroptosis of renal tubular epithelial cells based on TEM analysis, LDH measurement, and flow cytometry. These data support the notion that (1) necroptosis and necroinflammation are early events after ureteral ligation in UUO mice and (2) treatment with AKF-PD during the early phase blocks necroptosis and kidney injury, which may protect against renal fibrosis induced by UUO, but we could not conclude that exposure to AKF-PD could reverse the resulting fibrosis. Most importantly, we uncovered a possible mechanism for the antifibrotic effects of AKF-PD in UUO mice. Nevertheless, all of apoptosis, necroptosis, and ferroptosis have been found in IRI, folic acid–induced AKI, and UUO (Xiao et al., 2017; Popper et al., 2019; Linkermann et al., 2013; Ning et al., 2011; Oberbauer et al., 2001; Ortiz et al., 2000; Hu et al., 2019; Yang et al., 2020; Martin-Sanchez et al., 2018). We still could not completely exclude the influence of AKF-PD on apoptosis or ferroptosis from our *in vivo* data. As we know, the treatment of renal fibrosis is multi-targeted (Tampe and Zeisberg, 2014; Shi et al., 2020). So, AKF-PD may target multiple regulated cell death to protect against fibrosis. To illustrate it, further research using transgenic animals is warranted. Meanwhile, we also need to explore the effects of AKF-PD on necroptosis in more clinically relevant animal models.

Our UUO mouse model showed that a single injection of Nec-1 (inhibitor of RIPK1) ameliorated kidney injury, release of proinflammatory cytokines (IL-1β and TNF-α) and a chemokine (MCP-1), and renal fibrosis. Nec-1 is identical to methyl-thiohydantoin-tryptophan and has been found to inhibit indoleamine 2,3-dioxygenase (Vandenabeele et al., 2013). In addition, Nec-1 has been applied widely to investigate the contribution of RIPK1 in necroptosis and necroinflammation in various disease models in recent years (Mulay Shrikant et al., 2016; Wen et al., 2017; Shen et al., 2019). Use of Nec-1 in the present study suggested that necroptosis possibly participated in renal fibrosis in UUO mice, although we could not exclude the possible off-target effects of Nec-1 against indoleamine 2,3-dioxygenase. Therefore, more studies are needed to clarify the role of AKF-PD in transgenic animals to eliminate the off-target effect.

Inflammasomes are multiprotein complexes assembled by cytosolic “sensor” molecules (e.g., ASC, NLRs, and caspase-1). Inflammasomes mediate expression of inflammatory caspases to trigger pyroptotic cell death and regulate cleavage of proinflammatory cytokines (e.g., IL-1β and IL-18) into secreted and mature forms (Man and Thirumala-Devi, 2015). Activated caspase-1 processes and cleaves gasdermin D to induce pyroptosis. Recently, it has been reported that RIPK3 or MLKL also activated NLRP3-caspase-1 inflammasomes (Wang et al., 2014; Kang et al., 2015; Lawlor Kate et al., 2015). Our previous studies revealed that AKF-PD attenuated inflammation by inhibiting inflammasome activation, lowering expression of caspase-1, and thereby decreasing cleavage of pro-IL-1β into IL-1β (Song et al., 2016). However, the association among the inflammasome, pyroptosis, and RIPK3/MLKL pathway is not well known. The discovery that AKF-PD inhibits the RIPK3/MLKL pathway in the present study raises the question whether AKF-PD suppresses pyroptosis via RIPK3-, MLKL-, and NLRP3 inflammasome-dependent pathways.

## Conclusion

AKF-PD and Nec-1 exert effective activity against renal fibrosis, and AKF-PD may inhibit inflammation through RIPK3-mediated necroptosis. Our data may provide new targets and a novel treatment strategy against renal fibrosis.

## Data Availability Statement

All datasets generated for this study are included in the article.

## Ethics Statement

The animal study was reviewed and approved by the Ethics Review Committee for Animal Experimentation of Central South University.

## Author Contributions

QD and LH designed the research; LT guided the design of the research; QD, ZP, XHL, and YJ carried out the animal studies; XL, YZ, and XY performed the other studies; LT, JM, YX, QY, and ZP contributed new reagents or analytic tools; and QD and LH analyzed the data and wrote the manuscript.

## Funding

This study was supported by the National Natural Science Foundation of China Nos. 81600582 and 81900679.

## Conflict of Interest

The authors declare that the research was conducted in the absence of any commercial or financial relationships that could be construed as a potential conflict of interest.

## Abbreviations

AKF-PD, fluorofenidone; UUO, unilateral ureteral obstruction; DAMPs, damage-associated molecular patterns; CKD, chronic kidney disease; Nec-1, necrostatin-1; RIP: receptor-interacting protein kinase; MLKL: mixed lineage kinase domain-like protein; LDH: lactate dehydrogenase; TEM: transmission electron microscopy; HK-2: human renal tubular epithelial; TNF-α: tumor necrosis factor-alpha; MCP-1: monocyte chemoattractant protein-1
